# Classification and treatment of distal radius fractures: a survey among orthopaedic trauma surgeons and residents

**DOI:** 10.1007/s00068-016-0635-z

**Published:** 2016-02-12

**Authors:** M. A. M. Mulders, D. Rikli, J. C. Goslings, N. W. L. Schep

**Affiliations:** 1 Trauma Unit, Department of Surgery, Academic Medical Center, University of Amsterdam, Meibergdreef 9, 1105 AZ Amsterdam, The Netherlands; 2 Department of Surgery, University Hospital Basel, University of Basel, Spitalstrasse 21, 4056 Basel, Switzerland; 3 Department of Surgery, Maasstad Hospital, Maasstadweg 21, 3079 DZ Rotterdam, The Netherlands

**Keywords:** Distal radius, Survey, Fracture, Dislocated, Classification, Treatment

## Abstract

**Purpose:**

Classification, the definition of an acceptable reduction and indications for surgery in distal radius fracture management are still subject of debate. The purpose of this study was to characterise current distal radius fracture management in Europe.

**Methods:**

During the European Congress of Trauma and Emergency Surgery (ECTES) 2015 a 20-question multiple-choice survey was conducted among the attending surgeons and residents of the hand and wrist session. Consensus was defined as more than 50 % identical answers (moderate consensus 50–75 % and high consensus more than 75 %).

**Results:**

A total of 46 surgeons and residents participated in the survey. High consensus was found among both surgeons and residents for defining the AO/OTA classification as the preferred classification system. For the definition of an acceptable reduction, a moderate to high consensus could be determined. Overall, high consensus was found for non-operative treatment instead of operative treatment in dislocated extra- and intra-articular distal radius fractures with an acceptable closed reduction, regardless of age. We found high (surgeons) and moderate (residents) consensus on the statement that an intra-articular gap or step-off ≥2 mm, in patients younger than 65 years, is an absolute indication for ORIF. The same applied for ORIF in dislocated fractures without an acceptable closed reduction in patients younger than 75 years of age.

**Conclusion:**

Current distal radius fracture management in Europe is characterised by a moderate to high consensus on the majority of aspects of fracture management.

## Introduction

Despite the high incidence of distal radius fractures, around 20–32 per 10,000 person-year [1, 2], many aspects in distal radius fracture management remain a subject of debate. This is in particular true for a reliable and reproducible classification, the definition of an acceptable reduction and when to operate a patient with a distal radius fracture [3, 4].

First, until now around 20 different classification systems for distal radius fractures have been proposed. Several studies determined the intra- and inter-observer reliability for the most frequently used classification systems and all studies showed a low reproducibility and reliability [5–11]. Furthermore, it is questionable if these classification systems help to guide treatment and prognosis.

Second, another issue is the absence of a well-defined and validated definition of what constitutes an acceptable reduction. This is illustrated by the fact that studies and guidelines use different definitions of acceptable reduction [3, 4, 12–14]. This phenomenon is possibly caused by the contradicting evidence concerning the correlation between the quality of reduction and functional outcome [14–19].

Third, the optimal method of treatment of patients with distal radius fractures remains inconclusive. We know that non-dislocated fractures can be treated non-operatively, with good anatomical and functional results [12, 13, 20]. However, treatment of dislocated fractures remains a subject of debate [3, 4, 21]. Especially when closed reduction achieved an acceptable position, the question remains if these patients should be treated operatively. Two randomised controlled trials focused on this topic in the elderly [22, 23]. Both studies compared volar locking plate fixation with plaster immobilization in patients with an acceptable closed reduction and showed that the operative group had a better wrist function in the first 3 months, as indicated by lower DASH and PRWE scores. Nevertheless, after 1 year no differences were found. For the young and active population, however, it is undecided which treatment leads to the best functional outcome.

These debatable aspects may lead to practice variation. Additionally, previous studies showed that this practice variation could also be explained by the age and socioeconomic status of the patient, as well as the age and specialization of the surgeon [24–26]. In conclusion, this variation indicates a lack of clear evidence in distal radius fracture management [27]. To characterise current distal radius fracture management in Europe, a survey was conducted among the participants at an European Trauma and Emergency Congress.

## Methods

During the European Congress of Trauma and Emergency Surgery (ECTES) in Amsterdam in May 2015, a survey was conducted among the attending orthopaedic/trauma surgeons and residents of the hand and wrist session.

**Fig. 1:**
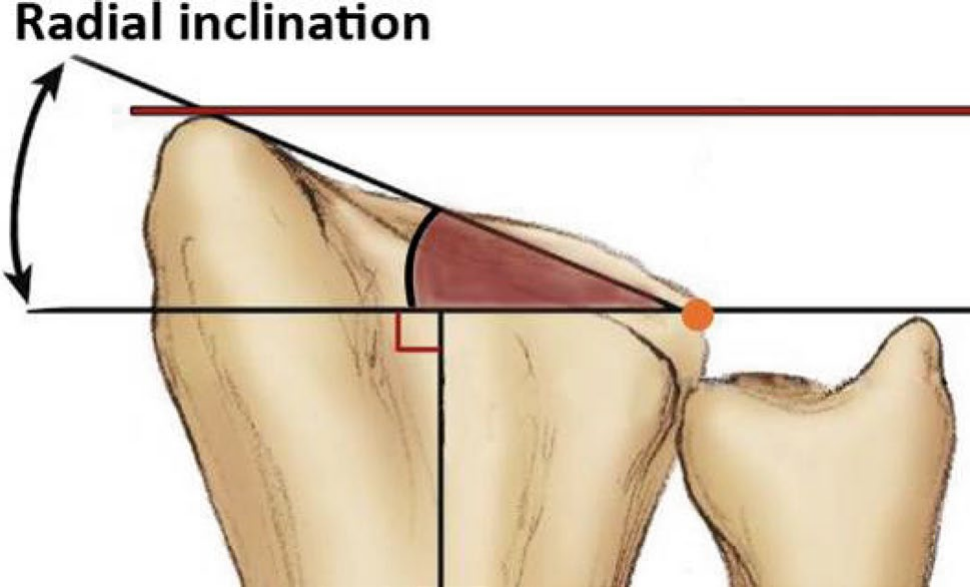
Radial inclination

**Fig. 2:**
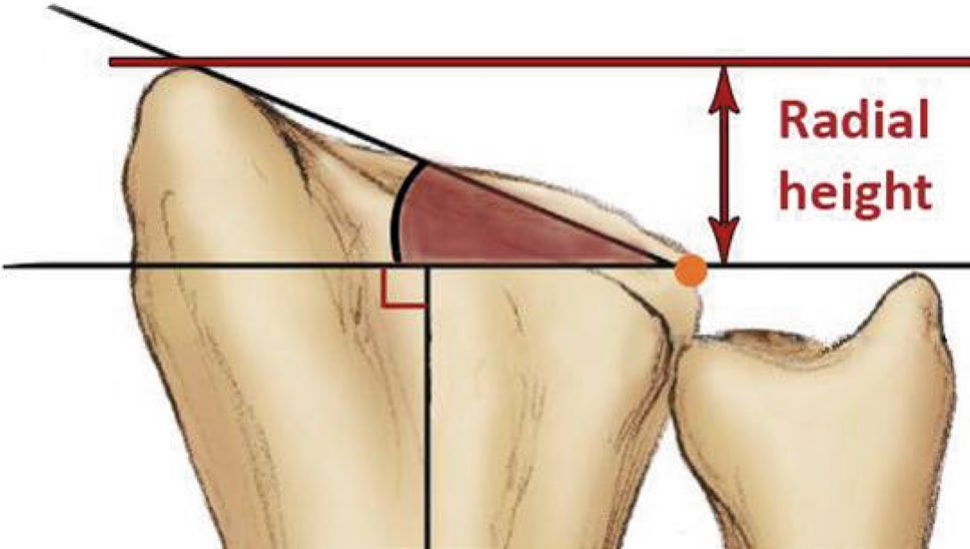
Radial height

**Fig. 3:**
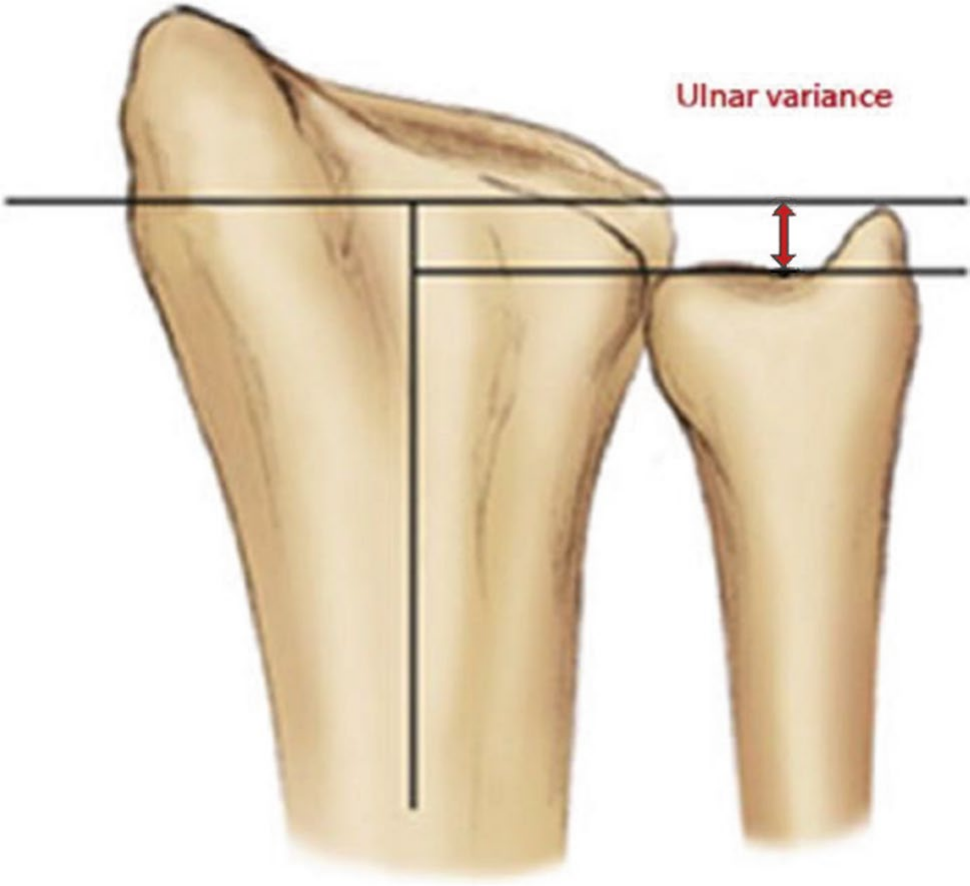
Ulnar variance

**Fig. 4:**
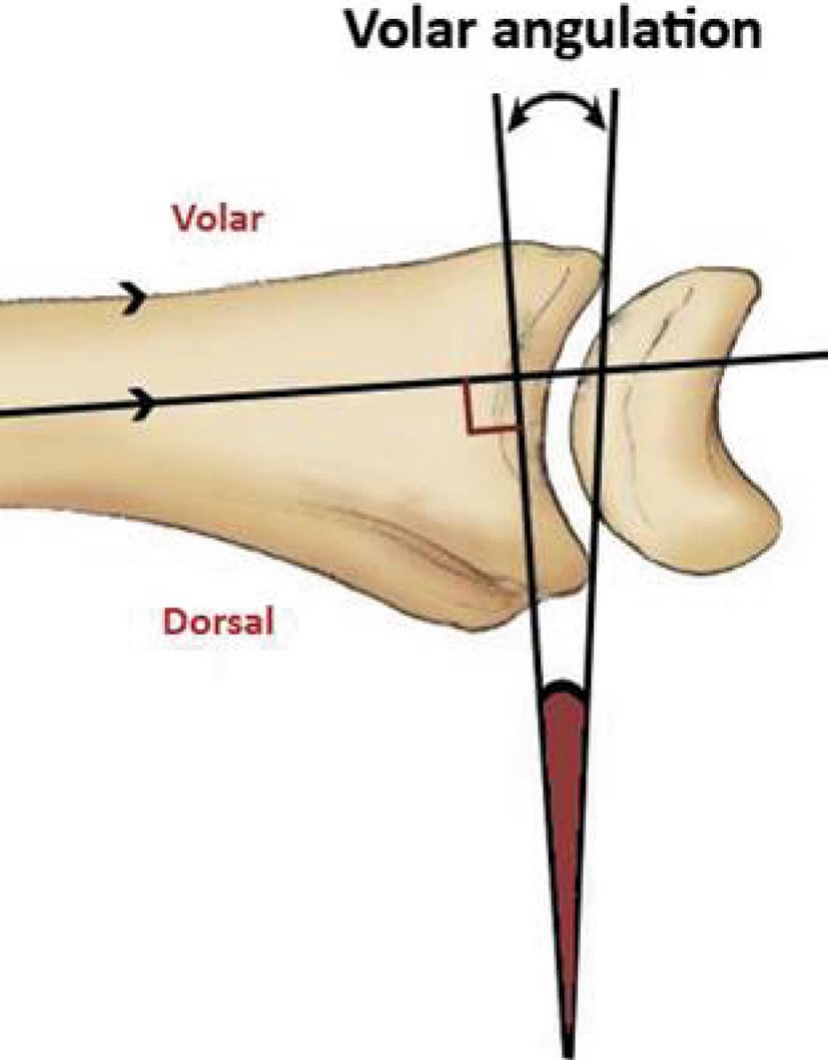
Volar angulation

**Fig. 5:**
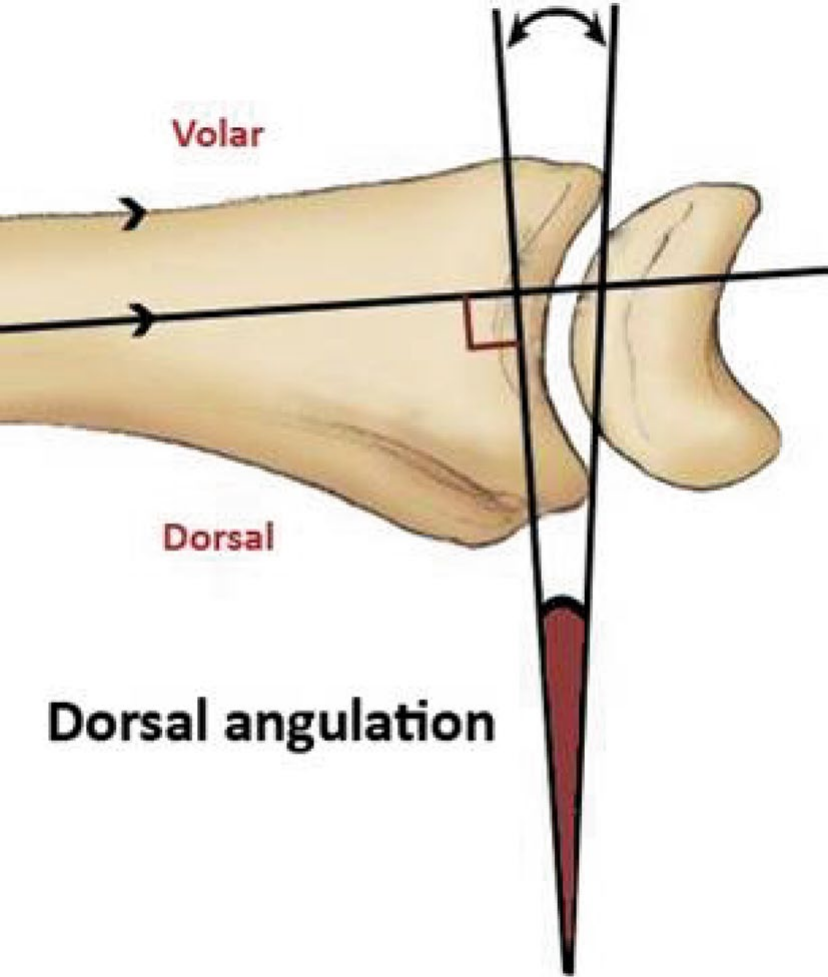
Dorsal angulation

**Fig. 6:**
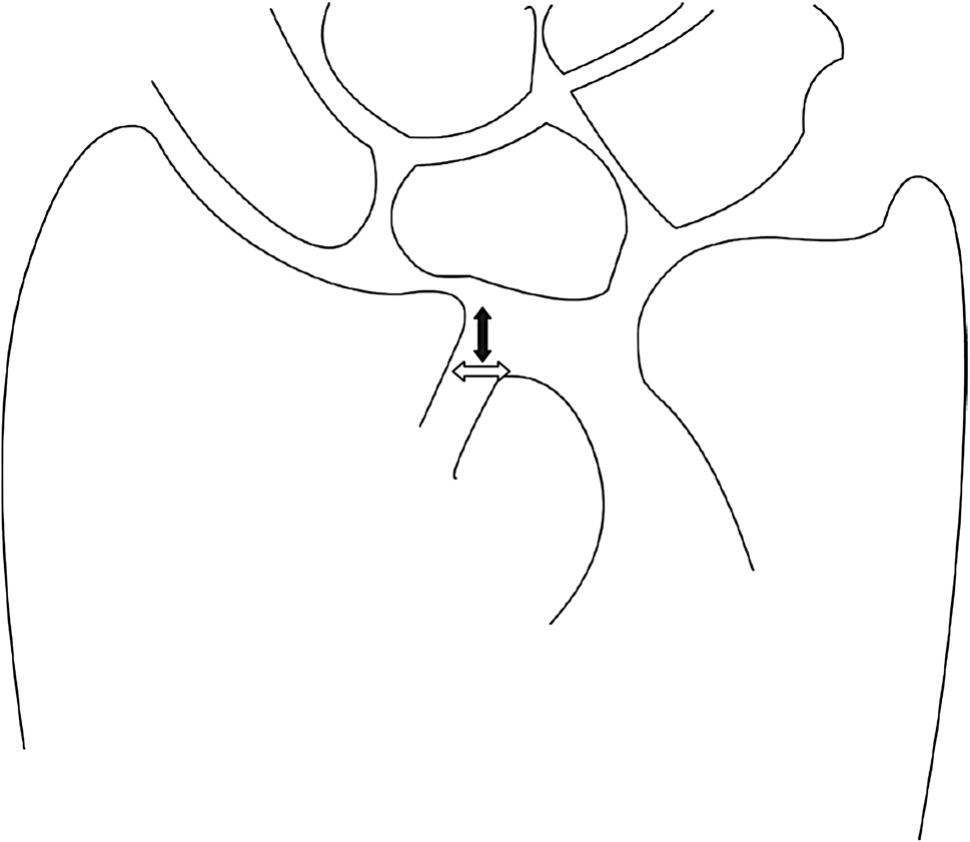
Intra-articular gap (*white arrow*) and step-off (*black arrow*)

A 20-question multiple-choice survey was designed to assess the differences in clinical practice patterns in Europe (“Appendix”). The survey consisted of three modules: classification of distal radius fractures, definition of an acceptable reduction and the preferred treatment of dislocated distal radius fractures with and without acceptable closed reduction. The first module regarding classification consisted of two questions concerning the most popular classification systems and if this classification guides treatment and prognosis. The second module concerning acceptable reduction consisted of six questions. The participants were asked about their definition of an acceptable radial inclination (Fig. 1), radial height (Fig. 2), ulnar variance (Fig. 3), volar angulation (Fig. 4), dorsal angulation (Fig. 5) and intra-articular step-off and gap (Fig. 6). Illustrative images, exemplifying the measurements were displayed simultaneously. The last module of the survey concerning treatment of dislocated fractures consisted of ten questions and was divided into four subsections: intra-articular fractures with a step-off or gap ≥2 mm, dislocated extra- and intra-articular fractures with an acceptable closed reduction and dislocated fractures without an acceptable closed reduction. An acceptable closed reduction was defined as what was previously judged as acceptable by the participant. Additionally, a subdivision based on age was made. The same questions were asked for patients younger than 65 years of age, patient between 65 and 75 years and patients older than 75 years.

The survey was conducted using TurningPoint 5 software (Turning Technologies, Youngstown, USA) and a remote response system. Directly following each question, the answers were shown on the screen in the lecture hall. No right or wrong answers were given to the participants.

For descriptive outcome analysis, the respondents were divided into two different groups: surgeons and residents. High consensus was defined as more than 75 % identical answers and a moderate consensus was defined as 50–75 % of surgeons and residents agreeing on the same classification, definition or treatment. Everything below 50 % was defined as no consensus.

## Results

A total of 33 surgeons and 15 residents participated in the survey. Seventeen of 33 (52 %) surgeons and 11 of 15 (73 %) residents completed the entire survey. From the participants who skipped a question, 53 % skipped only one question. Two surgeons skipped more than five questions and were excluded from the descriptive analyses. This resulted in a total of 46 respondents.

### Surgeons

The average of distal radius fracture cases treated each month varied between surgeons. Fifty-six percent of surgeons treated more than ten cases on average a month (Table 1).

**Table 1:** Average amount of distal radius fracture cases treated per month (% of total), *N* = 46

	Surgeons	Residents
0–5	23	53
6–10	19	20
11–15	23	0
16–20	23	0
>20	10	27

#### Classification of distal radius fractures

There was high consensus on the AO/OTA classification as the preferred classification. From the 27 surgeons who preferred the AO/OTA classification, 50 % of surgeons indicated that the AO/OTA classification guides treatment and prognosis. The remaining 13 surgeons answered that the AO/OTA classification guides only treatment (27 %) or neither treatment nor prognosis (23 %). Overall, half of the surgeons believed the classification systems of their choice guides treatment and prognosis (Table 2).

**Table 2:** Classification of distal radius fractures (% of total), *N* = 46

	Surgeons	Residents
Preferred classification		
AO/OTA	87	87
Melone	0	0
Frykman	6.5	6.5
Fernandez	0	0
Other	6.5	6.5
Guides treatment and prognosis		
Only treatment	30	7
Only prognosis	0	7
Treatment and prognosis	47	47
Nor treatment nor prognosis	23	40

#### Definition of an acceptable reduction

For the definition of an acceptable reduction, a moderate to high consensus was found for: radial inclination of ≥15° (58 %), a radial height of >5 mm (66 %), a volar and dorsal angulation of <15° (78 %) and <10° (80 %), respectively, and an intra-articular gap or step-off of <2 mm (83 %). There was no consensus on the definition of an acceptable ulnar variance. An ulnar variance of 0 mm was most often (38 %) indicated as acceptable (Table 3).

**Table 3:** Definition of an acceptable reduction (% of total), *N* = 46

	Surgeons	Residents
Radial inclination		
≥10°	23	20
≥15°	58	67
≥20°	19	13
Radial height		
>5 mm	66	43
>9 mm	34	57
Ulnar variance		
≥2 mm	24	50
>1 mm	21	25
0 mm	38	17
<1 mm	7	0
≤2 mm	10	8
Volar angulation		
<15°	78	73
<20°	22	27
<25°	0	0
Dorsal angulation		
<10°	80	67
<15°	10	33
<20°	10	0
Step-off and gap		
<1 mm	17	13
<2 mm	83	73
<3 mm	0	13

#### Treatment of dislocated distal radius fractures

##### Intra-articular gap and step-off

There was a high consensus among the surgeons on the statement that an intra-articular gap or step-off in a patient younger than 65 years is an absolute indication for open reduction and internal fixation (ORIF) (Table 4).

**Table 4:** Preferred treatment of dislocated distal radius fractures (% of total), *N* = 46

	Surgeons	Residents
Intra-articular gap or step-off ≥2 mm in patient <65 years absolute indication for ORIF		
I agree	80	60
I disagree	20	40
Extra-articular fracture with acceptable closed reduction (AO/OTA type A2 and A3)		
<65 years		
Plaster	83	87
ORIF	10	13
Pins and plaster	7	0
External fixation	0	0
65–75 years		
Plaster	90	93
ORIF	7	7
Pins and plaster	3	0
External fixation	0	0
>75 years		
Plaster	100	93
ORIF	0	7
Pins and plaster	0	0
External fixation	0	0
Intra-articular fracture with acceptable closed reduction (AO/OTA type C)		
<65 years		
Plaster	66	40
ORIF	34	60
Pins and plaster	0	0
External fixation	0	0
65–75 years		
Plaster	77	80
ORIF	23	13
Pins and plaster	0	7
External fixation	0	0
>75 years		
Plaster	93	100
ORIF	3.5	0
Pins and plaster	3.5	0
External fixation	0	0
Dislocated fracture without acceptable closed reduction		
<65 years		
Plaster	3.5	0
ORIF	93	100
Pins and plaster	3.5	0
External fixation	0	0
65–75 years		
Plaster	3.5	6.5
ORIF	78	87
Pins and plaster	15	6.5
External fixation	3.5	0
>75 years		
Plaster	40	71
ORIF	33	21
Pins and plaster	13.5	7
External fixation	13.5	0

##### Dislocated extra-articular distal radius fracture with an acceptable closed reduction

For dislocated extra-articular distal radius fractures (AO/OTA type A2 and A3) with an acceptable closed reduction, a high consensus was found for plaster immobilization as the preferred treatment for all patients younger than 75 years. All surgeons agreed that conservative treatment in patients older than 75 years is the preferred treatment.

Three surgeons decided on ORIF and two surgeons on pins and plaster as the preferred treatment for patients younger than 65 years. For the patients of 65–75 years, ORIF was preferred by two surgeons and pins and plaster by one surgeon. None of the surgeons would treat patients with an extra-articular fracture and an acceptable closed reduction with an external fixator (Table 4).

##### Dislocated complete articular distal radius fracture with an acceptable closed reduction

In patients older than 65 years with an initially dislocated complete articular fracture (AO/OTA type C) with an acceptable closed reduction, a high consensus was found for plaster immobilization as the preferred treatment. In patients younger than 65 years, a moderate consensus was found for plaster immobilization as the preferred treatment.

ORIF was the treatment of choice in patients younger than 65 years, in 34 % of surgeons. In patients from 65 to 75 years, ORIF was the preferred treatment for seven surgeons and for the elderly patients, aged above 75 years, two surgeons chose for, respectively, ORIF or pins and plaster. None of the surgeons would use an external fixator for patients with a complete articular fracture and an acceptable closed reduction (Table 4).

##### Dislocated distal radius fracture without an acceptable closed reduction

High consensus was found for ORIF in all patients younger than 75 years with a dislocated distal radius fracture without an acceptable closed reduction. Two surgeons favoured, respectively, plaster immobilization or pins and plaster in patients younger than 65 years. In patients of 65–75 years, 15 % of surgeons decided on treatment with pins and plaster and the minority (7 %) chose plaster immobilization or treatment with an external fixator.

However, for patients older than 75 years with a dislocated distal radius fracture without acceptable closed reduction, no consensus was found for the preferred treatment. Plaster immobilization was preferred in 40 % and operative treatment in 60 % of the surgeons (Table 4).

### Residents

The majority of the residents treat an average of zero to five distal radius cases per month (53 %). Twenty-seven percent of residents indicated that they treat more than 20 cases of distal radius fractures on average a month (Table 1).

#### Classification of distal radius fractures

Among the residents, high consensus was found for the AO/OTA classification as the preferred classification. However, from the 13 residents six residents (46 %) indicated that the AO/OTA classification guides treatment and prognosis and five residents (39 %) that it guides neither treatment nor prognosis. Overall, 47 % of the residents indicated that their preferred classification system guides treatment and prognosis and 40 % thinks it guides nor treatment nor prognosis (Table 2).

#### Definition of an acceptable reduction

For the definition of an acceptable reduction, a moderate consensus was found for: radial inclination of ≥15° (67 %), a radial height of >9 mm (57 %), a volar and dorsal angulation of <15° (73 %) and <10° (67 %), respectively, and an intra-articular gap or step-off of <2 mm (73 %). No consensus was found for the definition of an acceptable ulnar variance. An ulnar variance of ≥2 mm was most often (50 %) indicated as acceptable (Table 3).

#### Treatment of dislocated distal radius fractures

##### Intra-articular gap and step-off

A moderate consensus was found among the residents for the statement that an intra-articular gap or step-off in a patient younger than 65 years is an absolute indication for open reduction and internal fixation (ORIF) (Table 4).

##### Dislocated extra-articular distal radius fracture, with an acceptable closed reduction

For the initially dislocated extra-articular fracture (AO/OTA type A2 and A3) with an acceptable closed reduction, high consensus was found for plaster immobilization as the preferred treatment in all patients. Two residents chose ORIF for patients younger than 65 years and one resident for, respectively, patients between 65 and 75 years and patients older than 75 years. None of the residents chose treatment with pins and plaster or an external fixator for patients with an extra-articular fracture and an acceptable closed reduction (Table 4).

##### Dislocated complete articular distal radius fracture, with an acceptable closed reduction

For the initially dislocated complete articular fracture (AO/OTA type C) with an acceptable closed reduction, high consensus was found for plaster immobilization as the preferred treatment for patients from 65 to 75 years. All residents agreed on conservative treatment in patients older than 75 years. However, for patients younger than 65 years, moderate consensus was found for ORIF instead of plaster immobilization as the preferred treatment. In these patients, plaster immobilization was the treatment of choice in 40 %.

In patients from 65 to 75 years, ORIF was the preferred treatment by two residents and pins and plaster by one resident. None of the residents would treat these patients with a complete articular fracture and an acceptable closed reduction with external fixation (Table 4).

##### Dislocated distal radius fracture, without an acceptable closed reduction

All residents chose ORIF as the preferred treatment for a dislocated distal radius fracture without an acceptable closed reduction in patient 65 years or younger and a high consensus for patients from 65 to 75 years. However, for patients older than 75 years, a moderate consensus was found for plaster immobilization instead of ORIF as the preferred treatment for the dislocated fractures without an acceptable closed reduction.

In patients from 65 to 75 years, one resident chose treatment with pins and plaster and one resident chose nonoperative treatment with plaster immobilization. In the patients older than 75 years, four residents preferred operative treatment. Also for the dislocated fractures without an acceptable closed reduction, none of the residents decided on treatment with an external fixator (Table 4).

## Discussion

A high consensus was found in both surgeons and residents for the AO/OTA classification as the preferred classification system. However, only half of the surgeons and residents indicated that this classification system guides treatment and prognosis. Additionally, 39 % of the residents and 23 % of the surgeons even think it guides neither treatment nor prognosis. This lack of confidence in the classification system is supported by the literature and guidelines, which both state that there is need for a proper classification system which is more user-friendly, has a higher intra- and inter-observer reliability and which guides both treatment and prognosis [4, 10, 11, 28]. Although, CT-scanning of distal radius fractures is becoming more popular, it does not significantly improve the inter- and intra-observer agreement for most classification systems [29, 30].

In the literature, different definitions for an acceptable closed reduction are used. When assessing the definition of an acceptable closed reduction among the participants of this survey, a high consensus could be determined among surgeons for the definition of an acceptable volar and dorsal angulation and intra-articular gap or step-off. And a moderate consensus on the definition of a radial inclination and radial height (both surgeons and residents) and volar and dorsal angulation and intra-articular gap or step-off for residents. However, a difference was found between the surgeons and residents on the definition of an acceptable radial height, respectively >5 and >9 mm. Additionally, the definition of an acceptable ulnar variance provided a wide variety in answers. Although in literature ulnar variance is widely used to predict instability [12, 31] and radiographic alignment following operative or non-operative treatment of distal radius fractures [22, 32–34], our results question whether ulnar variance is as well-known as we think it is and how it is best used in distal radius fracture management. Moreover, there was consensus among both surgeons and residents on an intra-articular step-off and gap of less than 2 mm, indicating an acceptable reduction. However, this consensus is likely based on the study of Knirk and Jupiter [35], which was corrected in 2009 due to methodological flaws [36]. Therefore, we might conclude that this consensus is likely based on an opinion rather than on evidence.

Variability in treatment strategies for patients with dislocated distal radius fractures exists. However, in this survey there was a moderate to high consensus among both surgeons and residents on the indicated treatment strategies. In general, non-operative treatment was preferred over operative treatment in dislocated extra- and intra-articular distal radius fractures with an acceptable closed reduction, regardless of age. Only the residents preferred ORIF as treatment for complete articular distal radius fractures with an acceptable closed reduction in patients younger than 65 years, where surgeons would rather treat those patients with plaster immobilization.

In two recent randomised controlled trials [22, 23], operative treatment with a volar locking plate was compared with non-operative treatment with plaster immobilization in patient of 65 years or older with a dislocated distal radius fracture and an acceptable closed reduction. These studies showed that after 1 year there was no significant difference between these two groups regarding wrist function, represented by DASH and PRWE scores. Nevertheless, for younger patients, we await the results of the current VIPER trial, which compares the functional results of ORIF versus plaster immobilization for dislocated extra-articular distal radius fractures with an acceptable closed reduction in patients younger than 75 years [37].

The preferred treatment for patients younger than 65 years with intra-articular fractures with a gap or step-off ≥2 mm was ORIF, for both surgeons and residents. The same was true for dislocated fractures without an acceptable closed reduction in patients younger than 75 years of age. However, for the elderly patients above 75 years, plaster immobilization was favoured. This choice for nonoperative treatment is likely based on studies that show that elderly patients do not experience greater satisfaction or better functional outcomes when acceptable reduction is achieved [17, 38].

Although, surgeons and residents in our survey only differed on two aspects of treatment strategies for dislocated distal radius fractures, we can generally state that residents are more likely to prefer ORIF over non-operative treatment for the younger patients compared to surgeons. Additionally, none of the residents decided on treatment with an external fixator for dislocated distal radius fractures, regardless of quality of reduction. This finding corresponds with a recent study of Waljee et al. [25]. They found that younger surgeons were more likely to perform ORIF and significantly less likely to perform external fixation and percutaneous pinning for distal radius fractures. In addition, two recent meta-analysis showed that ORIF with a volar locking plate leads to significant better functional outcomes and lower DASH scores throughout the entire follow-up, compared to treatment with an external fixator [39, 40]. However, this significant difference was not clinically relevant after 3 months. Moreover, fewer complications have been identified in patients treated with ORIF, instead of external fixation or fixation with pins. Especially pin-track infections are avoided when using ORIF [41].

This study has some drawbacks. Due to the fact that we used a survey, response bias could have been present. First, the participants of this survey were able to see the responses after the polling was closed. Maybe this could have influenced their answer to the next question. Second, the participants were potentially able to discuss the answers to the questions with their colleagues or random people sitting next to them during the session. Also this could have led to response bias. Another possible limitation is that patients could have given a desirable answer, while in real practice they would have done something different. Additionally, we did not asked the nationality of the participants to see if there would be a difference in distal radius fracture management between different countries in Europe.

When determining the criteria for an acceptable closed reduction, we did not take into account the evaluation of the distal radio ulnar joint (DRUJ). Moreover, more factors are important in distal radius fracture management than only age and acceptable closed reduction. These factors include hand dominance, occupation, expectations of the patient and patient preferences, and all play an important role in decision-making.

To determine the different treatment strategies for the different fracture types, we only used the AO/OTA classification, because it is the most often used classification system in clinical setting. Though, not all clinicians use the AO/OTA classification, based on the answers in our survey.

Last, to our knowledge, a clear definition of consensus does not exist. Therefore, we arbitrarily defined high consensus as 75 % of participants agreeing on the same answer and moderate consensus of 50–75 % of participants giving on the same answer.

## Conclusion

There is a moderate to high European consensus on the majority of aspects of distal radius fracture management. A full consensus about distal radius fracture management will probably never be possible, because surgeons will always have their preferences and expert-based strategies [24, 42, 43]. Additionally patient preferences play an important role in fracture management. However, the remaining variability in answers given in this survey reflects the need for more well designed randomised controlled trials with a homogeneous patient population.

## Appendix

General information:

1. What is your profession?
  - Orthopaedic surgeon
  - Trauma surgeon
  - Fellow
  - Resident
  - (PhD) student
2. How many distal radius fracture cases do you treat in average within a month?
  - 0–5 cases
  - 6–10 cases
  - 11–15 cases
  - 16–20 cases
  - >20 cases
    Classification distal radius fractures:
3. Which classification for distal radius fractures do you use most of the time in practice?
  - AO/OTA classification
  - Melone classification
  - Frykman classification
  - Fernandez classification
  - Other
4. Do you think your preferred classification system guides treatment and prognosis?
  - Guides only treatment
  - Guides only prognosis
  - Guides treatment and prognosis
  - Guides nor treatment nor prognosis

What is the definition of an acceptable reduction:

- Radial inclination ≥10°
- Radial inclination ≥15°
- Radial inclination ≥20°
- Radial height >5 mm
- Radial height >9 mm
- Ulnar variance ≥2 mm
- Ulnar variance >1 mm
- Ulnar variance 0 mm
- Ulnar variance <1 mm
- Ulnar variance ≤2 mm
- Volar angulation <15°
- Volar angulation <20°
- Volar angulation <25°
- Dorsal angulation <10°
- Dorsal angulation <15°
- Dorsal angulation <20°
- Step-off and gap <1 mm
- Step-off and gap <2 mm
- Step-off and gap <3 mm

Treatment of distal radius fractures:

5. An intra-articular gap and step-off in a patient younger than 65 years is an absolute indication for open reposition and internal fixation:
  - I agree
  - I disagree
6. An initially dislocated extra-articular distal radius fracture (AO type A2 and A3), with acceptable closed reduction, in patients younger than 65 years do I treat preferably with:
  - Plaster immobilization
  - Open reduction and internal plate fixation
  - Pins and plaster
  - External fixation
7. An initially dislocated extra-articular distal radius fracture (AO type A2 and A3), with acceptable closed reduction, in patients 65–75 years do I treat preferably with:
  - Plaster immobilization
  - Open reduction and internal plate fixation
  - Pins and plaster
  - External fixation
8. An initially dislocated extra-articular distal radius fracture (AO type A2 and A3), with acceptable closed reduction, in patients older than 75 years do I treat preferably with:
  - Plaster immobilization
  - Open reduction and internal plate fixation
  - Pins and plaster
  - External fixation
9. An initially dislocated complete articular distal radius fracture (AO type C), with acceptable closed reduction, in patients younger than 65 years do I treat preferably with:
  - Plaster immobilization
  - Open reduction and internal fixation with a plate
  - Pins and plaster
  - External fixation
10. An initially dislocated complete articular distal radius fracture (AO type C), with acceptable closed reduction, in patients 65–75 years do I treat preferably with:
  - Plaster immobilization
  - Open reduction and internal fixation with a plate
  - Pins and plaster
  - External fixation
11. An initially dislocated complete articular distal radius fracture (AO type C), with acceptable closed reduction, in patients older than 75 years do I treat preferably with:
  - Plaster immobilization
  - Open reduction and internal fixation with a plate
  - Pins and plaster
  - External fixation
12. Dislocated distal radius fractures without acceptable closed reduction in patients of 65 years or younger, I treat preferably with:
  - Plaster immobilisation
  - Open reduction and internal fixation with a plate
  - Pins and plaster
  - External fixation
13. Dislocated distal radius fractures without acceptable closed reduction in patients of 65–75 years, I treat preferably with:
  - Plaster immobilisation
  - Open reduction and internal fixation with a plate
  - Pins and plaster
  - External fixation
14. Dislocated distal radius fractures without acceptable closed reduction in patients of 75 years or older, I treat preferably with:
  - Plaster immobilisation
  - Open reduction and internal fixation with a plate
  - Pins and plaster
  - External fixation
